# Prodrug-Based Targeting Approach for Inflammatory Bowel Diseases Therapy: Mechanistic Study of Phospholipid-Linker-Cyclosporine PLA_2_-Mediated Activation

**DOI:** 10.3390/ijms23052673

**Published:** 2022-02-28

**Authors:** Milica Markovic, Karina Abramov-Harpaz, Clil Regev, Shimon Ben-Shabat, Aaron Aponick, Ellen M. Zimmermann, Yifat Miller, Arik Dahan

**Affiliations:** 1 Department of Clinical Pharmacology, School of Pharmacy, Faculty of Health Sciences, Ben-Gurion University of the Negev, Beer-Sheva 8410501, Israel; milica@post.bgu.ac.il (M.M.); sbs@bgu.ac.il (S.B.-S.); 2 Department of Chemistry, Ben-Gurion University of the Negev, Beer-Sheva 8410501, Israel; karinaab@post.bgu.ac.il (K.A.-H.); regevcl@post.bgu.ac.il (C.R.); 3 Ilse Katz Institute for Nanoscale Science and Technology, Ben-Gurion University of the Negev, Beer-Sheva 8410501, Israel; 4 Department of Chemistry, University of Florida, Gainesville, FL 32603, USA; aaron.aponick@gmail.com; 5 Department of Medicine, Division of Gastroenterology, University of Florida, Gainesville, FL 32610, USA; ellen.zimmermann@medicine.ufl.edu

**Keywords:** inflammatory bowel disease, drug targeting, oral drug delivery, prodrug, cyclosporine, phospholipase A_2_

## Abstract

Therapeutics with activity specifically at the inflamed sites throughout the gastrointestinal tract (GIT) would be a major advance in our therapeutic approach to inflammatory bowel disease (IBD). We aimed to develop the prodrug approach that can allow such site-specific drug delivery. Currently, using cyclosporine as a drug of choice in IBD is limited to the most severe cases due to substantial systemic toxicities and narrow therapeutic index of this drug. Previously, we synthesized a series of a phospholipid-linker-cyclosporine (PLC) prodrugs designed to exploit the overexpression of phospholipase A_2_ (PLA_2_) in the inflamed intestinal tissues, as the prodrug-activating enzyme. Nevertheless, the extent and rate of prodrug activation differed significantly. In this study we applied in-vitro and modern in-silico tools based on molecular dynamics (MD) simulation, to gain insight into the dynamics and mechanisms of the PLC prodrug activation. We aimed to elucidate the reason for the significant activation change between different linker lengths in our prodrug design. Our work reveals that the PLC conjugate with the 12-carbon linker length yields the optimal prodrug activation by PLA_2_ in comparison to shorter linker length (6-carbons). This optimized length efficiently allows cyclosporine to be released from the prodrug to the active pocket of PLA_2_. This newly developed mechanistic approach, presented in this study, can be applied for future prodrug optimization to accomplish optimal prodrug activation and drug targeting in various conditions that include overexpression of PLA_2_.

## 1. Introduction

Inflammatory bowel diseases (IBD) are a group of chronic inflammatory diseases of the gastrointestinal tract (GIT) and include Crohn’s disease and ulcerative colitis [1]. In the past decade, these diseases have emerged as a public health challenge worldwide [2,3]. To date, therapeutic strategies in IBD are largely based on anti-inflammatory drugs, steroids, and biological therapy [4,5,6,7]. Due to severe side effects in some patients with IBD, these therapies eventually need to be discontinued [8].

Cyclosporine has been extensively studied and is well-known for its anti-inflammatory effects and immunosuppressive activity [8,9,10,11,12]. It has been used to treat refractory or severely active IBD [13,14]. Cyclosporine’s mechanism of action is binding to cyclophilin and blocking the phosphatase activity of calcineurin, which in turn inhibits T-cell mediated cytokine production [11,15]. The treatment with cyclosporine is restricted to the most severe cases due to substantial systemic toxicities and the narrow therapeutic index [16,17]. Despite numerous side effects of cyclosporine, the treatment with cyclosporine for short-term use in patients that are hospitalized with severely active ulcerative colitis is still maintained, due to its efficiency [8,15,18]. To circumvent the serious side effects, particularly for long-term treatment, there is a strong need for an alternative, safer drug delivery of cyclosporine and improved site targeting to minimize systemic exposure.

Expression and activity of phospholipase A_2_ (PLA_2_) enzyme is considerably increased in the inflamed intestinal tissues of patients with IBD [19,20,21,22,23]. This enzyme recognizes the *sn*-2 acyl bond of a phospholipid (PL) and catalytically hydrolyzes the bond, releasing arachidonic acid and lysophospholipid (LPL). In our previous work, we have developed a new drug targeting approach, a PL-based prodrug approach [24,25]. Most recently, we have developed a library of PL-prodrugs containing PL linked to the cyclosporine through an alkyl linker [26]. These prodrugs differ in the number of the CH_2_- units (i.e., the length of the linker). The synthesis of the phospholipid-linker-cyclosporine (PLC) prodrugs includes two step condensation of the PL to the cyclosporine through diacyl chloride linkers with diverse lengths [26]. In these PLC prodrugs the fatty acid within the *sn*-2 position of the PL is replaced by cyclosporine-linker moiety. This approach uses PLA_2_ as the prodrug-activating enzyme, that allows releasing of the free drug from the PLC complex. In this approach the significantly elevated levels of the enzyme specifically in the inflammation sites, allow release of the free cyclosporin from the PLC prodrug specifically, at the inflamed sites. Thus, this approach effectively targets the regions of intestinal inflammation.

This work elucidates mechanistic background of prodrug activation and dynamics. Our preliminary in-vitro studies demonstrated that the chemical link (carbonic linker) between the cyclosporine and the PL affects the level of recognition and activation by PLA_2_ [26]. In this work, we aimed to further explore the effect of different levels of the PLA_2_ enzyme on the activation of different PLC prodrugs. This finding might demonstrate the differences in the rate of PLC prodrugs in healthy vs. diseased tissues. Nevertheless, the exact mechanistic reasoning behind PLC activation by PLA_2_ remains unknown. In addition, by applying molecular modeling tools, we provide insights into the molecular mechanism and the interpretation of in-vitro results. The main finding from the joined in-silico/in-vitro studies, is that the PLA_2_-mediated activation of the prodrug highly depends on the prodrug structure and linker length. The best activation efficiency occurs for the 12- carbon linker PLC prodrug that binds effectively to the pocket of the PLA_2_. On the other hand, with the shorter PL-linker-cyclosporine prodrugs steric hindrance disrupts the prodrugs entry into the enzyme pocket.

The enzyme PLA_2_ plays an important role in the inflammation. It is responsible for releasing a free arachidonic acid from the PL, and initiating arachidonic acid metabolic pathway, and consequent synthesis of lipid inflammatory mediators, such as prostaglandins, thromboxanes and leukotrienes [27]. Taking this into account, we anticipate that this study can serve as a basis for use of cyclosporine prodrugs (or any other PL-based prodrugs), as well as control of their activation in several other conditions which include inflammation and thus, overexpression of PLA_2_ [28,29].

## 2. Results and Discussion

### 2.1. Design and Activation of PLA_2_-Trigerred PLC Prodrug Depends on the Length of the Linker

The traditional prodrug approach focuses on altering diverse physicochemical features of the parent drug by binding to the hydrophilic/lipophilic functional groups to enhance the solubility or the passive permeability of the drug [30,31]. Recent modern prodrug strategies are based on promoieties that are attached to the parent drug to target specific membrane transporters or enzymes [32,33]. To provide specific drug targeting, these strategies consider molecular or cellular parameters, such as membrane transporter influx/efflux, enzyme expression and distribution [34,35]. Some approaches utilize lipids, such as PL, as carriers [36]. Such prodrugs have several advantages. First, they can accompany the physiological lipid trafficking pathways [37]. Second, they could target the specific step in lipid processing, particularly if the pathway is changed in the disease. Third, they may facilitate drug release at the specific target site [38].

**Figure 1:**
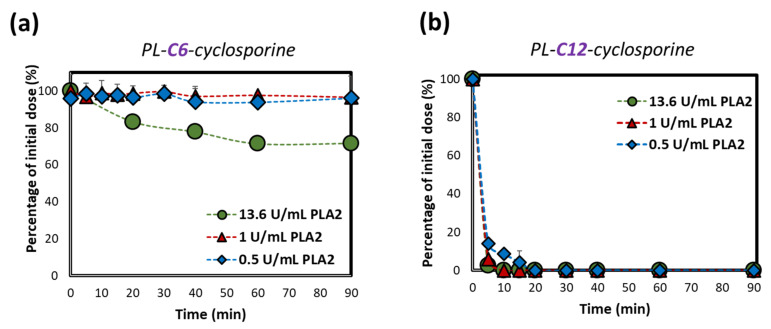
Activation rate (% of initial dose remaining) of PLC prodrugs that differ by the linker lengths, (**a**) 6-, and (**b**) 12-CH_2_ units, following incubation with 0.5, 1 and 13.6 U/mL bee venom PLA_2_. Data are presented as average ± SD; *n* = 3.

We have previously used this approach in the in-vitro proof-of-concept studies for PL-based prodrugs of diclofenac and indomethacin [24,25]. The structure of PLC prodrugs and preliminary in-vitro studies are described in our previous work [26]. The PLC designed prodrug consists of cyclosporine, bound to the sn-2 position of the PL through a linker that mimics the fatty acid substrate. The synthesis of four PLC prodrug is detailed in our previous work [26]. The NMR data specification of the 2 PLC prodrugs used in this study are seen in Figures S1 and S2. The in-vitro activation profile for three different concentrations of PLA_2_, was evaluated for both shortest and longest linker lengths (Figure 1). The PLC with the shorter linker length (6-CH_2_) demonstrated PLA_2_ concentration-dependent activation. Simultaneously PL-C6-cyclosporine lacks extensive PLA_2_-mediated hydrolysis (following the incubation in the solution) with the lowest PLA_2_ hydrolysis at 0.5 U/mL concentration. At concentrations above 1 U/mL of PLA_2_, PLA_2_-mediated activation is higher. Longer linker length, PL-C12-cyclosporine, resulted in complete, rapid hydrolysis that was entirely independent on the concentration of the PLA_2_.

In summary, the PLA_2_ hydrolysis of the PLC prodrugs showed clear evidence that the linker length is crucial for the ability of the enzyme to hydrolyze the ester bond. It was also shown that the extent of hydrolysis is highly dependent upon the concentrations of the PLA_2_ enzyme. This confirms our hypothesis that enzyme over-expression in the inflamed tissues will selectively activate the prodrug, as opposed to low concentrations of the enzyme in the healthy tissues.

### 2.2. Insights into the Molecular Mechanisms of the Activation of the PLC Prodrug

To provide insights and interpretation into the prodrug activation, molecular dynamics (MD) simulations were performed for two PLC prodrugs, (1) PL-C6-cyclosporine and (2) PL-C12-cyclosporine, were bound to the pocket of PLA_2_ enzyme. The specific binding to the PLA_2_ relies on the *sn*-2 acyl bond of the PLC conjugates and surrounding water molecules that play a role in the enzymatic hydrolysis. Therefore, the interaction in the pocket occurs between the oxygen atom from the prodrug carbonyl group and His34 residue within PLA_2_ that is activated by surrounding water molecules.

**Figure 2:**
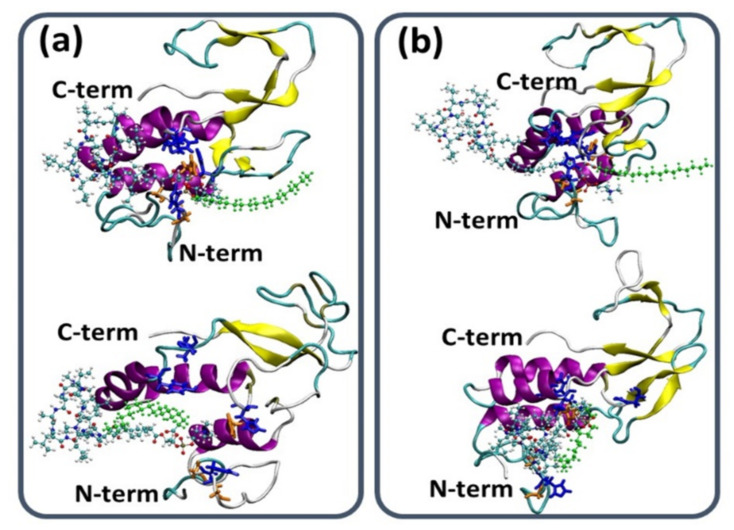
(**a**) Initial (top) and final simulated (bottom) model of the PL-C6-cyclosporine prodrug-PLA_2_ complex. (**b**) Initial (top) and final simulated (bottom) model of the PL-C12-cyclosporine prodrug-PLA_2_ complex. The hydrophobic chain in the *sn*-1 position of PL-prodrugs are colored in green. The hydrophobic chain of the PL-C6-cylosporine prodrug blocks the binding pocket of the PLA_2_. The opposite is true for the hydrophobic chain of the PL-C12-cylosporine, whose longer linker length allows access to the binding pocket. The illustration of the structures was performed by the vmd program [40].

The simulations demonstrate that the shorter, PL-C6-cyclosporine linker prodrug has been embedded into the binding site pocket of the PLA_2_, thus blocking the activation and drug liberation (Figure 2a). The longer, PL-C12-cyclosporine linker prodrug was exposed to the solution for the entire duration of the simulations. Hence, the longer linker (12-CH_2_) did not allow the binding site to be blocked with the long fatty acid chain in the *sn*-1 position; it is evident that the PL-C12-prodrug can easily contact the binding site pocket and consequently the activation occurs (Figure 2b). It is important to note that it is not the fatty acid in the sn-1 position, and the nature of this chain that plays a role in the activation, but rather the length of the linker in the sn-2 position. Previously, it was shown that His34 residue of the PLA_2_ enzyme is part of the binding site in the PLA_2_ [39].

**Figure 3:**
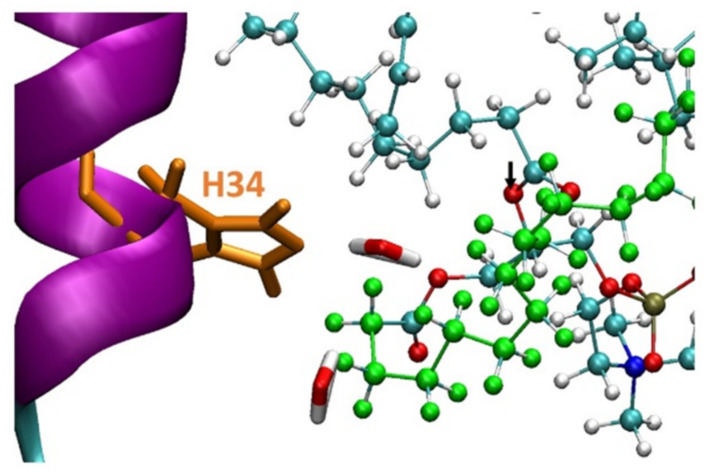
The His34 residue within the PLA_2_ at the close proximity to the active oxygen atom (seen in black arrow) in the PL-C12-cyclosporine prodrug. The hydrophobic chain of PL-prodrugs is colored in green. Two water molecules in close proximity to the His34 can also be observed. The illustration of the structures were performed by the vmd program [40].

**Figure 4:**
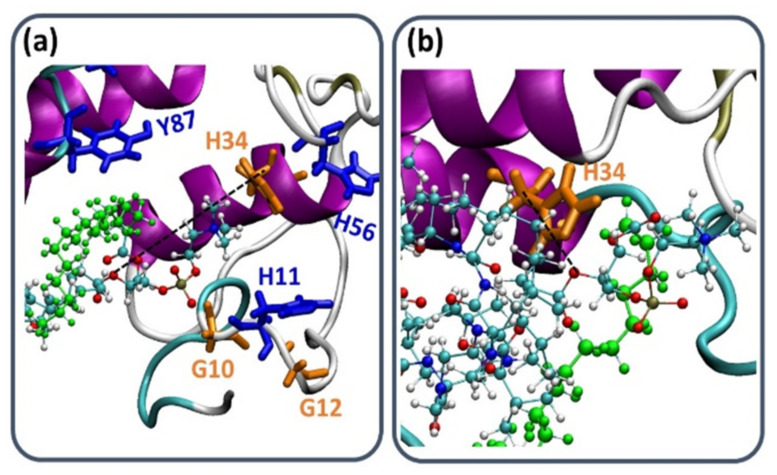
The distance between Cα of His34 and the active oxygen atom in (**a**) PL-C6-cyclosporine-PLA_2_ complex and (**b**) PL-C12-cyclosporine-PLA_2_ complex, showed that His34 is more in close proximity to the active oxygen in presence of PL-C12-cyclosporine than in presence of PL-C6-cyclosporine (Figure 5). The illustration of the structures were performed by the vmd program [40].

**Figure 5:**
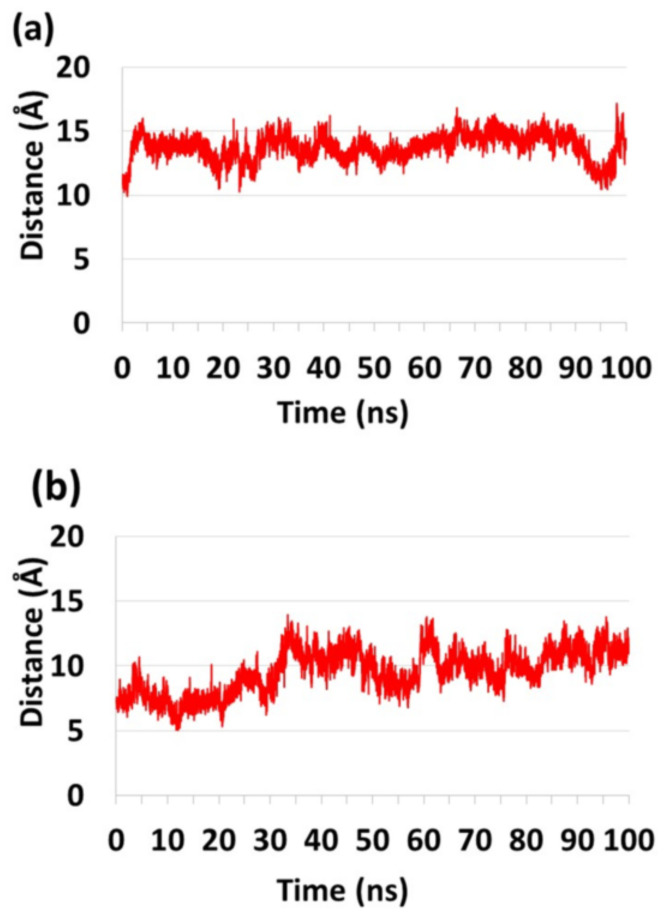
The distance between the Cα of His34 in PLA_2_ and the oxygen atom of the prodrugs (**a**) PL-C6-cyclosporine and (**b**) PL-C12-cyclosporine.

**Figure 6:**
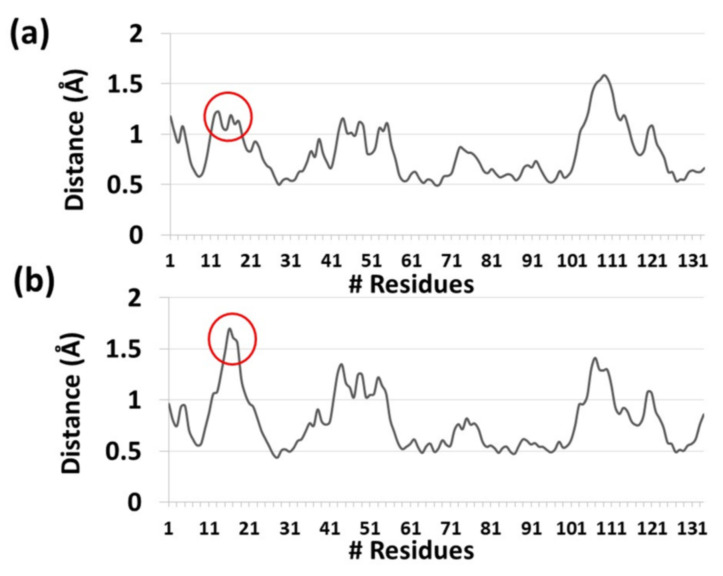
The root-mean-square fluctuations (RMSF) of each residue within PLA_2_ for the prodrugs (**a**) PL-C6-cyclosporine and (**b**) PL-C12-cyclosporine.

**Figure 7:**
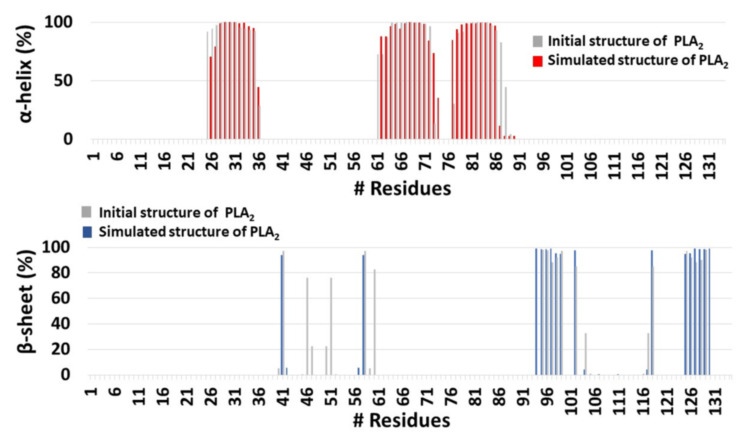
The helical and the β-sheet structures of PLA_2_ in the initial and simulated PL-C6-cyclosporine-PLA_2_ complex, using the database of secondary structure of protein (DSSP) method.

**Figure 8:**
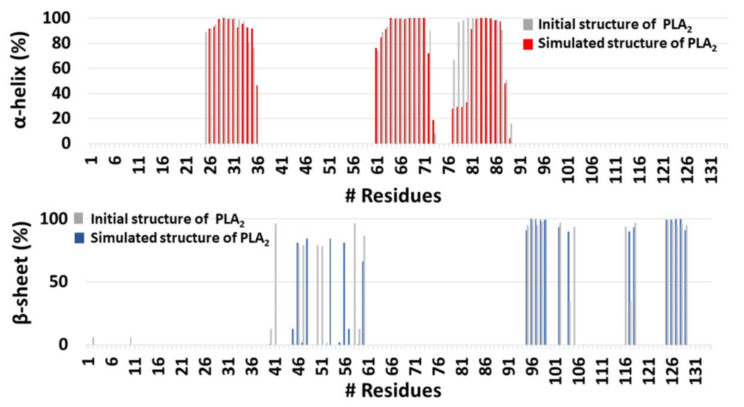
The helical and the β-sheet structures of PLA_2_ in the initial and simulated PL-C12-cyclosporine-PLA_2_ complex, using the database of secondary structure of protein (DSSP) method.

It was proposed that His34 probably operates as a Brønsted base and plays crucial role in the deprotonation of a water molecule [39]. Therefore, the His34 is responsible for the nucleophilic attack on the acyl bond. Indeed, our MD simulations showed that His34 is a crucial residue that plays a role in the activation of the PL-prodrug molecules, as well as water molecules which were observed in the proximity of the binding site pocket (Figure 3). The PL-C12-cyclosporine prodrug is completely exposed to the binding pocket of the PLA_2_. The fatty acid in the *sn*-1 position of the PL-C12-cyclosporine allows the active site and the His34 residue of the enzyme to interact with the desired active oxygen atom in the *sn*-2 position of the prodrug. This phenomenon does not occur with the PL-C6-cyclosporine prodrug in the binding pocket of the PLA_2_. To evaluate this phenomenon, the distance between the His34 and the active oxygen atom was measured for each one of the two complexes: PL-C6-cyclosporine- PLA_2_ and PL-C12-cyclosporine- PLA_2_ (Figure 4 and Figure 5). The N-terminal domain (residues 10–20) of PLA_2_ in PL-C6-cyclosporine-PLA_2_ complex is close to the prodrug PL-C6-cyclosporine, and to other domains in the PLA_2_, thus the N-terminal fluctuates less (Figure 6). In the PL-C12-cyclosporine-PLA_2_ complex, the fluctuation in the N-terminal domain (residues 10–20) is dramatically increased, due to the lack of the interactions with the PL-C12-cyclosporine and other domains in the PLA_2_ (Figure 6). The longer carbonic linker in the *sn*-2 position of the PL-C12-cyclosporine does not allow the N-terminal domain to interact with the prodrug. This result may explain the activation efficiency of the longer PL-C12-cyclosporine linker compared to the shorter PL-C6-cyclosporine. Finally, it is of interest to examine whether the secondary structure of the PLA_2_ is changed due to the interactions with the prodrugs. The simulations revealed that the helices of the PLA_2_ were conserved in the two prodrugs (Figure 7 and Figure 8). Interestingly, the β-strands along residues 45–60 within the PLA_2_ in the PL-C6-cyclosporine-PLA_2_ complex were disrupted, while in the PL-C12-cyclosporine-PLA_2_ complex the β-strands along these residues were conserved (Figure 7 and Figure 8).

In summary, the mechanism of this linker length effect on the prodrug activation pattern depends on the steric hindrance. The PLA_2_-mediated activation of the prodrug is highly relying on the prodrug structure, i.e., the spatial arrangement of the drug. This activation depends also on the fitting of the prodrug into the transition state geometry of PLA_2_, which dictates the binding between the prodrug and the enzyme. In fact, it was previously proposed that PLA_2_ only hydrolyses PL when the *sn*-2 position is occupied by fatty acid [41]. We have shown, however, that this is true when the drug is linked directly to the *sn*-2 position [42]. However, with the proper spacer between the PL and the drug moiety, the enzymatic activation can eventually occur [24,25,43]. It is important to note that for PL-based prodrugs of smaller drugs, such as, diclofenac and indomethacin, the short 6-carbon linker was found to be optimal, while both shorter and longer linkers inhibits/disrupts the prodrug activation [24,25]. Therefore, the optimal molecular design of PL-based prodrugs is depending on the size, the volume and the three-dimensional assembly of the specific drug.

Importantly, a great clinical advantage is offered by our drug targeting approach: the inflammation localization varies in IBD patients, and since up to date IBD drug products target a general intestinal region, these products will not be effective if the inflammation is outside the targeted region. Since our approach exploits a feature innate to the inflamed tissue(s) per-se (i.e., PLA_2_ overexpression), efficient treatment of any localization throughout the GIT is possible. Additionally, extended therapeutic index of clinically significant drugs may be achieved, maximizing cyclosporine levels in the inflamed intestinal tissues while minimizing systemic immunosuppression, thereby making our IBD targeting approach valuable in the search for improved drug therapy and overall patient care.

## 3. Materials and Methods

### 3.1. Materials

PLC prodrugs were synthesized using two step condensation of the PL to the cyclosporine through di-acyl chloride linkers with diverse lengths (6 and 12 methylene units); all steps required for structure elucidation and purity were completed (Figures S1 and S2). Phospholipase A_2_ from honey bee venom (*Apis mellifera*) was purchased from Sigma-Aldrich (Rehovot, Israel). Isopropanol, methanol, and water (Merck KGaA, Darmstadt, Germany) were of ultra-performance liquid chromatography (UPLC) grade. All other chemicals were of analytical reagent grade.

### 3.2. PLA_2_-Mediated Activation

PLA_2_ hydrolysis assay of the four newly synthesized PLC conjugates were carried out by bee venom PLA_2_ followed a previously published protocol with minor modifications [25,26]. Briefly, PLC conjugates with 6- and 12-carbon linker were dissolved in methanol, and small aliquots were added in 1 mL of buffer solution. Prior to prodrug addition, the buffer solutions with different concentrations of the bee venom PLA_2_ (0.5, 1 and 13.6) units/mL were made. The buffer solutions also contained Tris-HCl 10 mM, CaCl_2_ 10 mM and NaCl 300 mM (pH 7.4) with addition of 10mM sodium taurocholate, as a natural surfactant; the control group contained everything but bee venom PLA_2_. This mixture was incubated for 1.5 h at 25 °C. Samples were collected in intervals after 0, 5, 10, 15, 20, 30-, 40-, 60- and 90-min. Results for PLC activation are presented as mean ± SD; *n* = 4, per each phospholipid-cyclosporine conjugate.

### 3.3. Analytical Methods

Activation of PLC by different levels of PLA_2_ was followed by high performance liquid chromatography (HPLC) system (Waters 2695 Separation Module, Milford, MA, USA) with a photodiode array UV detector (Waters 2996, Milford, MA, USA). Separation of conjugates was performed with a C8 column and confirmed by UV. The HPLC conditions were as follows: Waters (WT186003055) Xbridge^®^ RP8 3.5 μm; 4.6 mm × 150 mm column, an isocratic mobile phase containing isopropanol: methanol: water (70:3:27 *v*/*v*) for 10 min at the flow rate of 0.5 mL/min and the detection wavelength was 206 nm.

### 3.4. Computational Modeling

#### 3.4.1. Parametrization and Integration of the Prodrugs

The integration of the two PLC prodrugs into the Chemistry at Harvard Macromolecular Mechanics (CHARMM) force field were performed by defining the parameters for the new molecules based on the well-established chemical analogs that were provided by CHARMM36 force-field (mackerell.umaryland.edu). According to the corresponding analog, each atom within the PLC prodrugs were defined by the charge, bond length, angles, torsion and van der Waals value. The assignments of the values were created by CGenFF generator: cgenff.umaryland.edu. The values demonstrated a reasonable deviation (less than 10% for all atoms within the PLC prodrugs).

#### 3.4.2. Construction of PL-C6-Cyclosporine and PL-C12-Cyclosporine Prodrugs

The coordinates for the PL-C6-cyclosporine and PL-C12-cyclosporine prodrugs that are complexed with the PLA_2_ enzyme were constructed using the Accelrys Discovery Studio software package (http://accelrys.com/products/discovery-studio/, accessed on 3 February 2022). Initial structure of the enzyme PLA_2_ applied in this work is the crystal structure of bee-venom PLA_2_ (PDB ID code: 1POC) [44]. It is crucial to accurately construct the initial structures of the two models representing the prodrug-enzyme complex. Therefore, the constructions of the models are based on previous experimental reports. It was proposed by experimental study that His34 residue of the PLA_2_ enzyme is part of the binding activated site in the PLA_2_ [39]. Moreover, it was proposed that His34 operates as a Brønsted base and plays crucial role in the deprotonation of a water molecule [39]. Hence, we constructed the prodrug-enzyme complex in which the prodrug acyl group of each PLC was inserted into the binding pocket in proximity to His34, while avoiding atom clashes. Specifically, each prodrug was inserted into the binding pocket of the enzyme in a similar manner: the shorter and the longer linkers were orientated in the same direction and the activated part of the prodrug *sn*-2 acyl group was oriented towards the binding pocket of the enzyme. It must be noted that there is only one possible option to make the modeling of these complexes, by producing a similarity in the positions of the linkers. Moreover, it is crucial that the linkers are in the same orientation, while the prodrug is being inserted into the binding pocket. Finally, we explored all possibilities for the constructions of these complexes, while keeping the modeling of the complexes in accordance to the experimental data, while keeping in mind the interactions of the prodrugs in the binding pocket of the enzyme. It is important to note that the constructions of the initial complexes did not account constrains, not in the construction modeling step nor along the molecular dynamics (MD) simulations.

#### 3.4.3. Molecular Dynamics (MD) Simulations Protocol

The MD simulations of the solvated constructed models were performed in the NPT ensemble using NAMD package [45] with the CHARMM27 forcefield with the CMAP correlation [46]. The energies of the complex prodrug-enzyme were minimized, and the model was explicitly solvated in a TIP3P water box [47,48]. Each water molecule within 2.5 Å of the models was removed. Counter ions were added at random locations to neutralize the models’ charge. The Langevin piston method [44,45,49] with a decay period of 100 fs and a damping time of 50 fs was used to maintain a constant pressure of 1 atm. The temperature 330 K was controlled by a Langevin thermostat with a damping coefficient of 10 ps [45]. The short-range van der Waals (VDW) interactions were calculated using the switching function, with a twin range cutoff of 10.0 and 12.0 Å. Long-range electrostatic interactions were calculated using the particle mesh Ewald method with a cutoff of 12.0 Å [50,51]. The equations of motion were integrated using the leapfrog integrator with a step of 1 fs. The counter ions and water molecules were allowed to move. The hydrogen atoms were constrained to the equilibrium bond using the SHAKE algorithm [52]. The minimized solvated systems were energy minimized for 5000 additional conjugate gradient steps and 20,000 heating steps at 250 K, with all atoms allowed to move. Then, the system was heated from 250 K to 300 K and then to 330 K for 300 ps and equilibrated at 330 K for 300 ps. The choice of the higher temperature than physiological temperature is to investigate the stability of the constructed models. Obviously, structures that are stable at higher temperature will be also stable at physiological temperature. Simulations ran for 100 ns for each variant model. To justify the timescale of the simulations, we computed the root-mean-square-deviation (RMSD) values along the MD simulations (Figure S3). The RAMS analysis demonstrated that after ~30 ns of the simulations, both variant models were converged. The structures were saved every 10 ps for analyses.

#### 3.4.4. Structural Analyses

The structural stabilities of the two models were measured using several analyses. The root-mean-square-fluctuation (RMSF) values for each residue within the PLA_2_ were computed for each model. To estimate possible interactions within the binding pocket of the PLA_2_ to the PLC prodrugs, the distances between specific Cα atoms of residues in the binding pocket and the oxygen atom in the *sn*-2 position were computed along the MD simulations. Finally, to examine whether the PLC prodrugs affect the secondary structure of the PLA_2_, the database of secondary structure of protein (DSSP) method has been applied [53]. This method was applied to provide the percentage of the α-helix or β-strand for each residue within the phospholipase along the MD simulations.

## 4. Conclusions

In this work we employed modern in-silico tools to confirm our experimentally obtained data, and to mechanistically explain the reasoning behind the significantly different activation rate among longer and shorter linkers in the PLC. We demonstrated that the PL-linker-cyclosporine with the longest linker exhibited optimal, fastest rate of activation through in-vitro PLA_2_-mediated activation, at all 3 enzyme concentrations. Consequently, we tested these results using the MD simulations, and provided mechanistic insights into the molecular mechanism activation of the PL-linker-cyclosporine with longer and shorter linkers. We have shown by MD simulations that the insufficient activation of the shorter PL-linker-cyclosporine is due to the steric hindrance that eventually does not appear in the longest PL-linker-cyclosporine. Ultimately, these studies can serve as a screening tool for optimal prodrug design, which can offer a modern biopharmaceutical solution for numerous clinical needs. The overexpression of PLA_2_ occurs in other malignant and inflammatory conditions, i.e., rheumatoid arthritis, colorectal cancer, and vascular inflammation [29,54]. Hence, our prodrug approach and mechanistic screening tools may offer an elegant solution for improving drug treatment for such diseases.

## Supplementary Materials

The following supporting information can be downloaded at: https://www.mdpi.com/article/10.3390/ijms23052673/s1.
