# Supplementary material for: Prodrug-Based Targeting Approach for Inflammatory Bowel Diseases Therapy: Mechanistic Study of Phospholipid-Linker-Cyclosporine PLA2-Mediated Activation
Source: Int J Mol Sci. 2022 Feb 28;23(5):2673. doi: 10.3390/ijms23052673 (PMC8910962; doi:10.3390/ijms23052673)
Supplement: Supplementary file 1 [file ijms-23-02673-s001.zip › ijms-1554479-supplementary.pdf]

Supporting Information

Prodrug approach for inflammatory bowel diseases: mechanistic study of phospholipid-linker-cyclosporine PLA_2-mediated activation

Milica Markovic[1], Karina Abramov-Harpaz[2,3], Clil Regev[2,3], Shimon Ben-Shabat[1], Aaron Aponick[4], Ellen M. Zimmermann[5], Yifat Miller[2,3,*], and Arik Dahan[1,*]

[1]*Department of Clinical Pharmacology, School of Pharmacy, Faculty of Health Sciences, Ben-Gurion University of the Negev, Beer-Sheva 8410501, Israel*

[2]*Department of Chemistry, Ben-Gurion University of the Negev, Beer-Sheva, 84105, Israel*

[3]*Ilse Katz Institute for Nanoscale Science and Technology, Ben-Gurion University of the Negev, Beer-Sheva 84105, Israel*

[4]*Department of Chemistry, University of Florida, Gainesville, FL 32603, USA*

[5]*Department of Medicine, Division of Gastroenterology, University of Florida, Gainesville, FL 32610, USA*

---

*Correspondences: Arik Dahan, Department of Clinical Pharmacology, School of Pharmacy, Faculty of Health Sciences, Ben-Gurion University of the Negev, Beer-Sheva 8410501, Israel. E-mail: arikd@bgu.ac.il; Yifat Miller, Department of Chemistry, Ilse Katz Institute for Nanoscale Science and Technology, Ben-Gurion University of the Negev, Beer-Sheva 8410501, Israel; E-mail: ymiller@bgu.ac.il

Data specification for PL-C6-cyclosporine and PL-C12-cyclosporine prodrug

PL-C6-cyclosporine (*cyclosporine-8-oxooctanoic acid-PL-conjugate*/5a): ^{1}H NMR (500 MHz, CDCl3) δ 8.55 (d, *J* = 10.0 Hz, 1H), 8.02 (d, *J* = 7.0 Hz, 1H), 7.48 (t, *J* = 7.0 Hz, 2H), 5.65 (dd, *J* = 11.0, 4.0 Hz, 1H), 5.48 (dd, *J* = 16.0, 11.5 Hz, 2H), 5.37 (dd, *J* = 12.0, 4.0 Hz, 1H), 5.29 – 5.07 (m, 5H), 4.94 – 4.90 (m, 2H), 4.82 (p, *J* = 7.0 Hz, 1H), 4.75 (t, *J* = 9.5 Hz, 1H), 4.62 (d, *J* = 14.0 Hz, 1H), 4.44 – 4.28 (m, 4H), 4.10 (dd, *J* = 12.0, 7.0 Hz, 1H), 4.00 – 3.88 (m, 2H), 3.80 – 3.77 (m, 2H), 3.44 (s, 3H), 3.36 (s, 9H), 3.21 (s, 6H), 3.19 (s, 3H), 3.08 (s, 3H), 3.00 – 2.90 (m, 3H), 2.64 (d, J = 9.0 Hz, 6H), 2.45 – 2.36 (m, 1H), 2.30 – 2.23 (m, 5H), 2.22 – 2.02 (m, 5H), 2.01 – 1.78 (m, 4H), 1.69 (t, *J* = 7.5 Hz, 2H), 1.64 – 1.50 (m, 12H), 1.47 – 1.39 (m, 2H), 1.39 – 1.19 (m, 45H), 1.03 – 0.90 (m, 20H), 0.88 – 0.74 (m, 30H); 13C NMR (151 MHz, CDCl3) δ 173.9, 173.7, 173.5, 173.4, 173.1, 173.0, 172.9, 171.5, 171.4, 170.9 (2 peaks at 170.998, 170.996), 170.4, 169.9, 168.2, 129.2, 126.5, 73.2,

70.7, 70.5, 66.7, 66.6 (2 peaks at 66.69, 66.67), 63.5 (2 peaks at 63.54, 63.50), 63.1, 59.4, 59.3, 58.4, 57.2, 56.4, 55.3, 54.8, 54.7 (2 peaks at 54.713, 54.710), 54.5, 50.1, 48.9, 48.4, 48.0, 44.7, 41.0, 39.3 (2 peaks at 39.376, 39.374), 37.1, 35.9, 34.4, 34.2, 34.0, 33.9, 33.2, 32.4, 32.0 (2 peaks at 32.047, 32.046), 31.8, 31.4, 30.2, 29.9, 29.8 (3 peaks at 29.84, 29.83, 29.80), 29.7 (3 peaks at 29.78, 29.75, 29.70), 29.6, 29.5, 29.4, 29.3, 29.2, 29.1, 25.0, 24.9, 24.8 (2 peaks at

24.88, 24.81), 24.7 (2 peaks at 24.79, 24.76), 24.4, 24.3, 24.1, 23.9, 23.8, 23.6, 22.8 (2 peaks at 22.814, 22.812), 21.9, 21.4, 21.2, 20.5, 19.7, 18.7, 18.3, 18.1, 17.8, 17.7, 15.1, 14.2, 10.0. HRMS (ESI) calcd for $C_{96}H_{175}N_{12}O_{21}P$ [M+Na]+ 1886.2625, found 1886.2577.

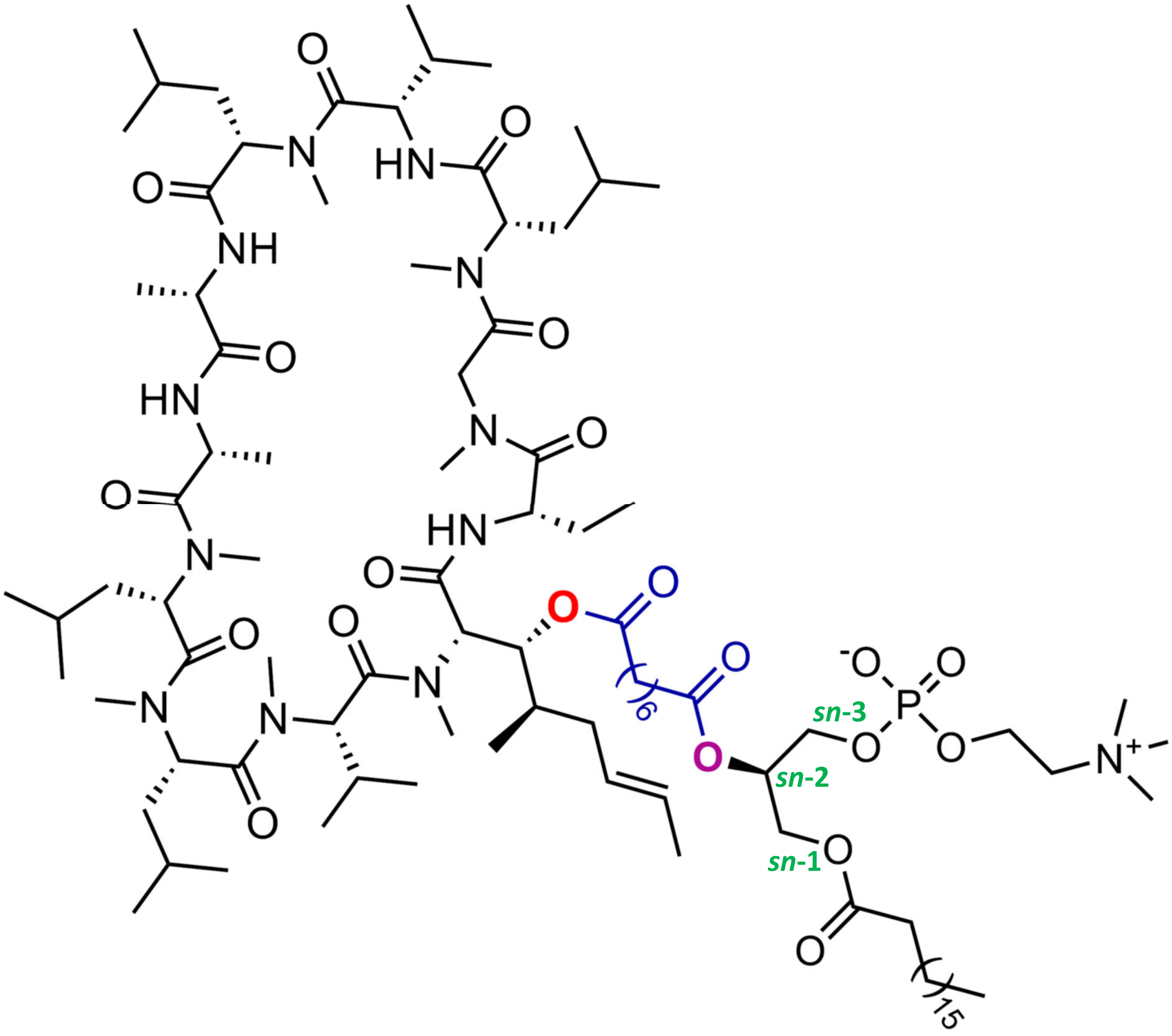

Figure S1a. Chemical structure of PL-C6-cyclosporine (cyclosporine-8-oxooctanoic acid-PL-conjugate/5a). Published in [1].

(A)

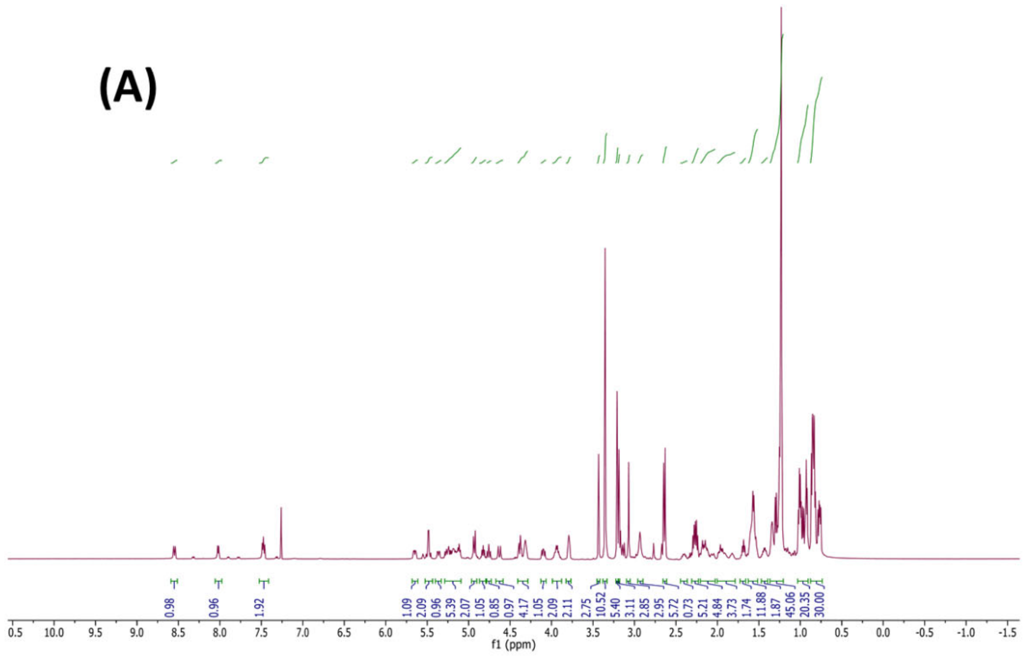

(B)

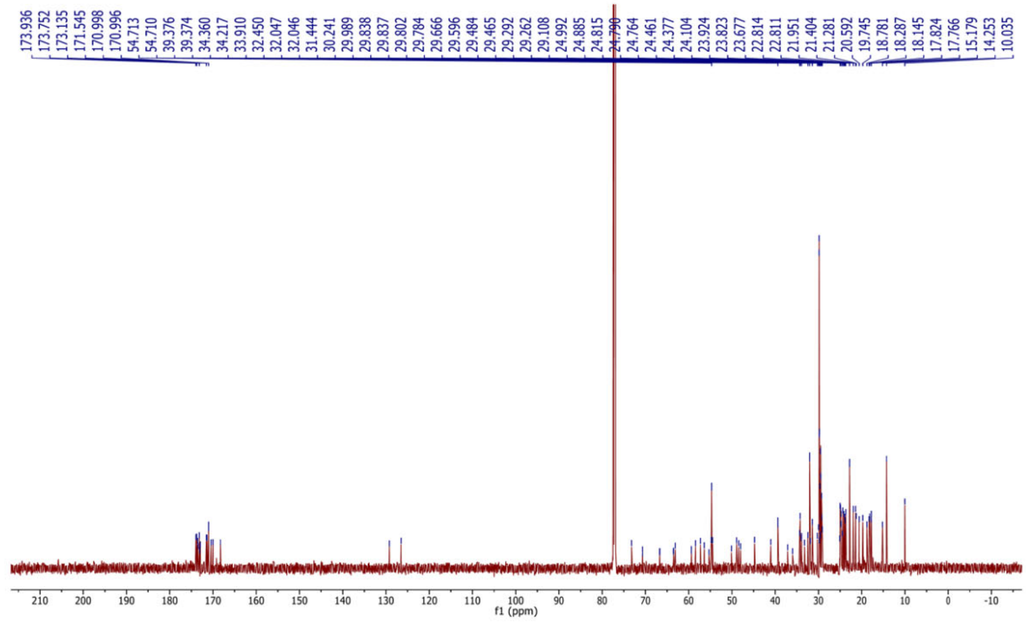

Figure S1b. NMR data for compound PL-C6-cyclosporine (cyclosporine-8-oxooctanoic acid-PL-conjugate/5a): (A) ^{1}H NMR (500 MHz, $CDCl_3$); (B) ^{13}C NMR (151 MHz, $CDCl_3$). Published in [1].

PL-C12-cyclosporine (*cyclosporine-14-oxotetradecanoic acid-PL-conjugate*, 5d), 1H NMR (600 MHz, CDCl3) δ 8.56 (d, *J* = 9.6 Hz, 1H), 8.02 (d, *J* = 6.6 Hz, 1H), 7.48 (t, *J* = 7.2 Hz, 2H), 5.66 (dd, *J* = 11.4, 4.2 Hz, 1H), 5.48 (dd, *J* = 28.5, 11.4 Hz, 2H), 5.38 (dd, *J* = 12.0, 3.6 Hz, 1H), 5.30 – 5.08 (m, 5H), 4.98 – 4.93 (m, 2H), 4.83 (p, *J* = 7.2 Hz, 1H), 4.76 (t, *J* = 9.6 Hz, 1H), 4.64 (d, *J* = 13.8 Hz, 1H), 4.42 – 4.26 (m, 4H), 4.11 (dd, *J* = 12.0, 7.2 Hz, 1H), 4.00 – 3.90 (m, 2H), 3.80 – 3.75 (m, 2H), 3.44 (s, 3H), 3.36 (s, 9H), 3.21 (d, *J* = 2.4 Hz, 6H), 3.19 (m, 3H), 3.08 (s, 3H), 2.84 – 2.74 (m, 3H), 2.64 (d, *J* = 7.2 Hz, 6H), 2.44 – 2.35 (m, 1H), 2.34 – 2.24 (m, 5H), 2.24 – 2.12 (m, 5H), 2.02 – 1.80 (m, 4H), 1.74 – 1.52 (m, 14H), 1.48 – 1.40 (m, 2H), 1.36 – 1.21 (m, 55H), 1.05 – 0.90 (m, 20H), 0.88 – 0.74 (m, 29H); 13C NMR (151 MHz, CDCl3) δ 173.9, 173.7, 173.5, 173.4, 173.3, 173.0, 171.6, 171.4, 171.0, 170.9, 170.4, 170.4, 169.8, 168.3, 129.3, 126.4, 73.1, 70.7, 70.6, 66.7 (3 peaks at 66.74, 66.71, 66.70), 63.5, 63.4, 63.1, 59.4, 59.3, 58.4, 57.3, 56.5, 55.3, 54.8, 54.7 (2 peaks at 54.75, 54.74), 54.5, 50.1, 48.9, 48.4, 48.0, 44.7, 41.0, 39.3 (2 peaks at 39.393, 39.391), 37.1, 36.0, 34.5, 34.3, 34.2, 33.9, 33.2, 32.5, 32.0, 31.9, 31.5, 31.4, 30.2, 29.9, 29.9, 29.8 (4 peaks at 29.84, 29.838, 29.836, 29.818) 29.7 (5 peaks at 29.791, 29.790, 29.743, 29.739, 29.706), 29.6 (2 peaks at 29.65, 29.61), 29.5 (2 peaks at 29.546, 29.545), 29.4 (2 peaks at 29.485, 29.452) 29.3 (2 peaks at 29.34, 29.30), 25.1 (3 peaks at 25.14, 25.12, 25.10), 25.0, 24.9, 24.8, 24.7, 24.4, 24.3, 24.0, 23.9, 23.8, 23.7, 22.8, 21.9, 21.4, 21.3, 20.5, 19.7, 18.7, 18.3, 18.1, 17.8, 17.7, 15.1, 14.2, 10.0; HRMS (ESI) calculated for $C_{102}H_{187}N_{12}O_{21}P$ [M+Na]+1970.3575, found 1970.3702.

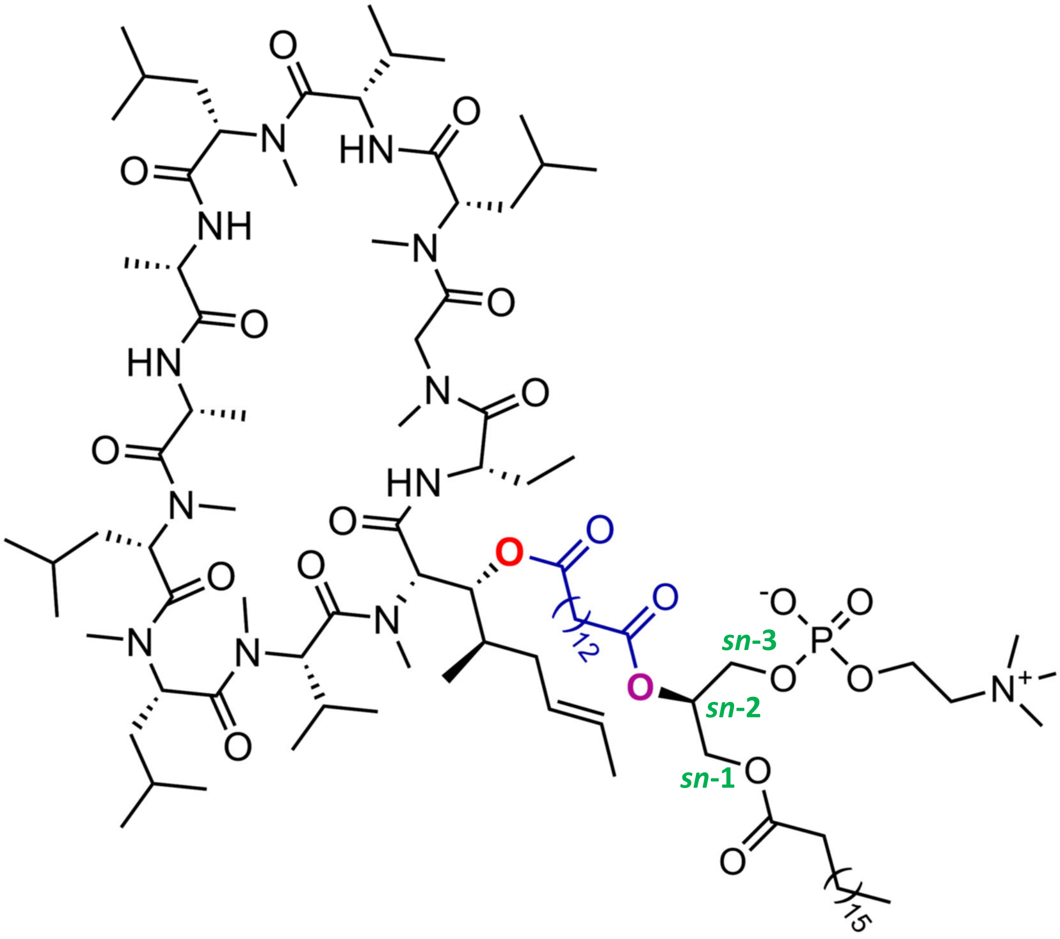

Figure S2a. Chemical structure of PL-C12-cyclosporine (cyclosporine-14-oxotetradecanoic acid-PL-conjugate, 5d). Published in [1].

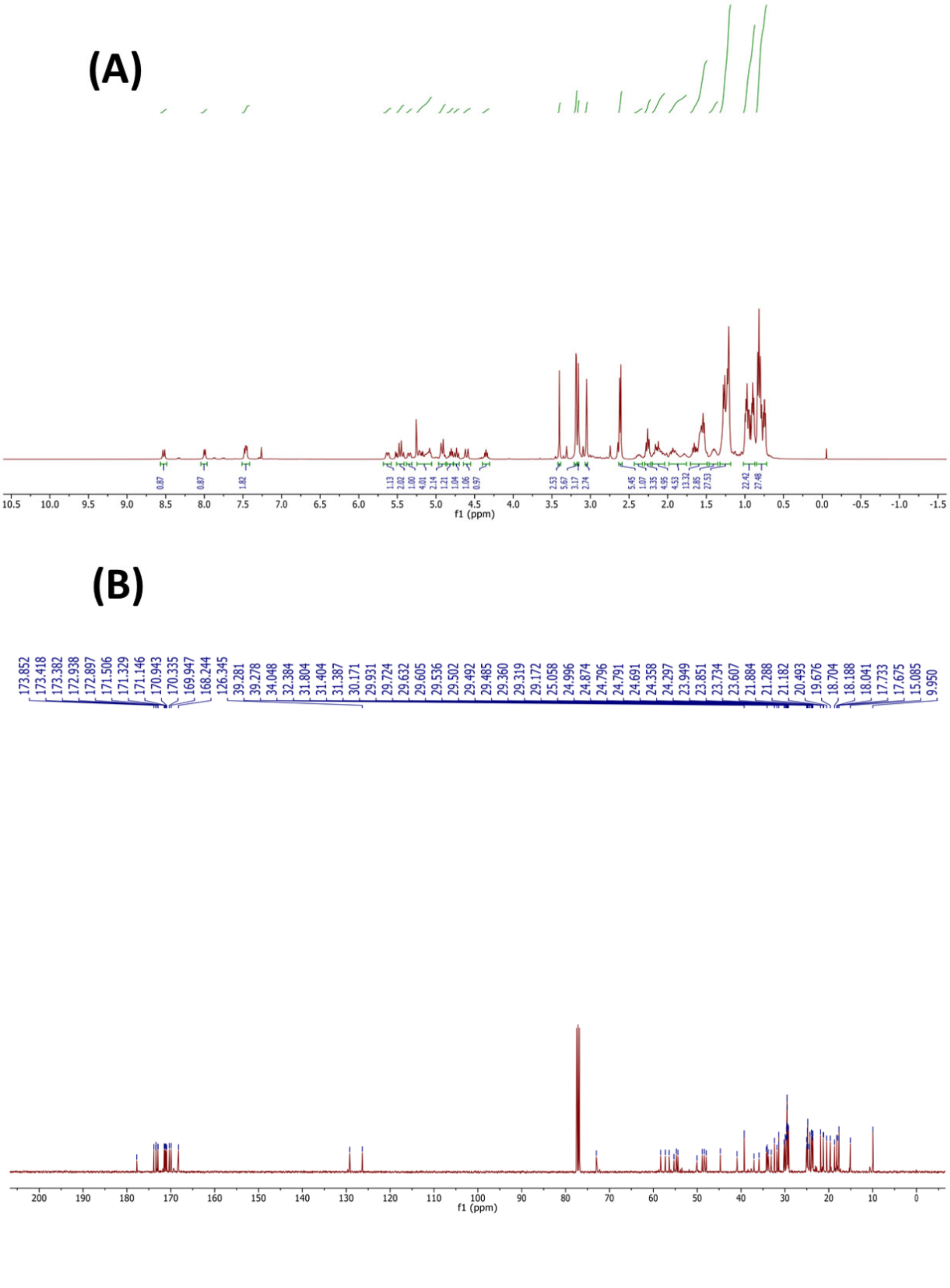

Figure S2b. NMR data for compound PL-C12-cyclosporine (cyclosporine-14-oxotetradecanoic acid-PL-conjugate, 5d): (A) ^{1}H NMR (500 MHz, $CDCl_3$); (B) ^{13}C NMR (151 MHz, $CDCl_3$). Published in [1].

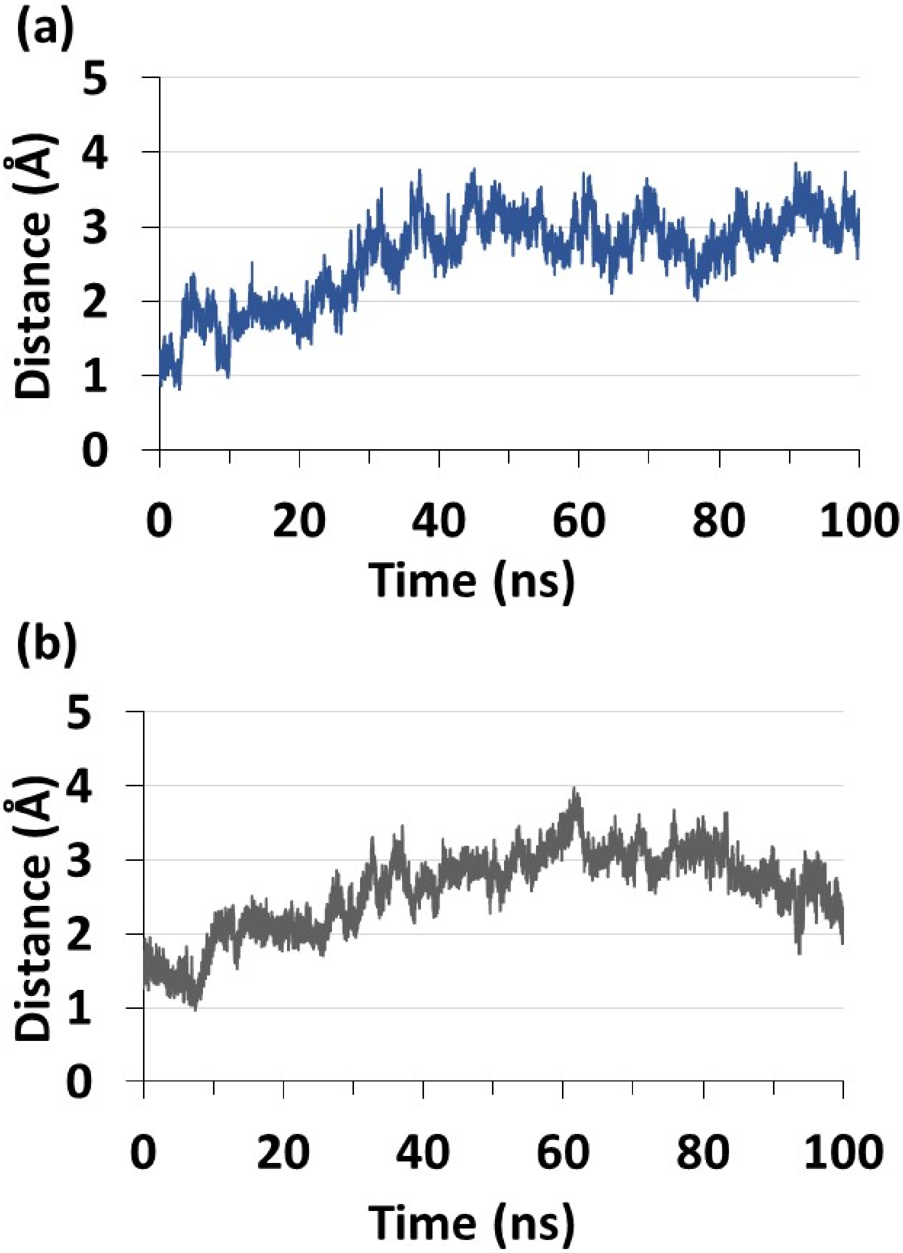

Figure S3. The root-mean-square-deviation (RMSD) values along the MD simulations for (a) PL-C6-cyclosporine prodrug-PLA_2 complex, and (b) PL-C12-cyclosporine prodrug-PLA_2 complex.

References

1. Manda, J.N.; Markovic, M.; Zimmermann, E.M.; Ben-Shabat, S.; Dahan, A.; Aponick, A. Phospholipid Cyclosporine Prodrugs Targeted at Inflammatory Bowel Disease (IBD) Treatment: Design, Synthesis, and in Vitro Validation. *ChemMedChem* **2020**, 10.1002/cmdc.202000317, doi:10.1002/cmdc.202000317.
